# A Lubricated Nonimmunogenic Neural Probe for Acute Insertion Trauma Minimization and Long‐Term Signal Recording

**DOI:** 10.1002/advs.202100231

**Published:** 2021-06-03

**Authors:** Yeontaek Lee, Hyogeun Shin, Dongwon Lee, Sungah Choi, Il‐Joo Cho, Jungmok Seo

**Affiliations:** ^1^ School of Electrical and Electronic Engineering Yonsei University Seoul 03722 Republic of Korea; ^2^ Center for BioMicrosystems Brain Science Institute Korea Institute of Science and Technology (KIST) Seoul 02792 Republic of Korea; ^3^ Division of Bio‐Medical Science & Technology, KIST School Korea University of Science and Technology (UST) Seoul 02792 Republic of Korea; ^4^ Yonsei‐KIST Convergence Research Institute Yonsei University Seoul 03722 Republic of Korea

**Keywords:** bio inspired surface, brain‐machine interfaces, neural probe, nonimmunogenic properties

## Abstract

Brain‐machine interfaces (BMIs) that link the brain to a machine are promising for the treatment of neurological disorders through the bi‐directional translation of neural information over extended periods. However, the longevity of such implanted devices remains limited by the deterioration of their signal sensitivity over time due to acute inflammation from insertion trauma and chronic inflammation caused by the foreign body reaction. To address this challenge, a lubricated surface is fabricated to minimize friction during insertion and avoid immunogenicity during neural signal recording. Reduced friction force leads to 86% less impulse on the brain tissue, and thus immediately increases the number of measured signal electrodes by 102% upon insertion. Furthermore, the signal measurable period increases from 8 to 16 weeks due to the prevention of gliosis. By significantly reducing insertion damage and the foreign body reaction, the lubricated immune‐stealthy probe surface (LIPS) can maximize the longevity of implantable BMIs.

## Graphical abstract

Signal sensitivity of neural probes deteriorates over time due to acute and chronic inflammations. To overcome these problems, lubricated immune‐stealth probe surface (LIPS) is fabricated with nonimmunogenic properties. Near‐frictionless properties of LIPS minimizing insertion trauma increases signal‐to‐noise ratio and the number of neurons from short‐term neural recording. Furthermore, anti‐biofouling properties of LIPS improve its longevity, doubling the signal recording time‐length.

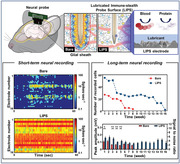

## 1 Introduction

Brain‐machine interfaces (BMIs) have been spotlighted as a tool to help people with extensive clinical disabilities.^[^ ^1^ ^]^ For example, recorded brain signals using chronically implanted devices can be decoded into a synthesized voice^[^ ^2^ ^]^ and can control the movement of computer cursors^[^ ^3^ ^]^ and robotic limbs.^[^ ^4^ ^]^ Therefore, it is essential to seamlessly integrate the interface between the brain tissue and the implanted device to record and control neurons over long periods. In recent decades, many neural probes with different shapes,^[^ ^5^ ^]^ materials,^[^ ^6^ ^]^ and multiplexities^[^ ^7^ ^]^ have been developed for firm integration with brain tissue to record neural signals stably from neurons. However, implanted probes for clinical BMIs have not yet been widely adopted, mainly because of the biological inflammatory response (e.g., gliosis) around the probe‐tissue interface that arises from acute insertion trauma and chronic foreign body reaction. Acute insertion trauma not only causes an increase of acute neural loss but also accelerates the initial formation of the glial sheath around the implanted probe.^[^ ^8^ ^]^ Furthermore, the glial sheath thickness increases gradually due to the foreign body reaction, eventually driving neurons away from the electrodes beyond the effective recording range.

To address the issue of gliosis progression, there have been attempts to make probes flexible or combine drug‐eluting coatings on their surfaces. The aim of flexible neural probes is to reduce the chronic immune response resulting from the shearing micromotion between the probe material and the neural tissue. Softer material properties in flexible probes have led to less mechanical strain on the surrounding tissue and a minimal chronic immune response resulting from micromotion.^[^ ^9^ ^]^ A drug‐eluting coating containing anti‐inflammatory drugs has been explored most often as it has the potential to reduce glial encapsulation.^[^ ^10^ ^]^ However, flexible neural probes with drug‐eluting coatings also come with limitations such as insertion difficulty due to their ultraflexibility. To enable the insertion of flexible probes into the brain, insertion force has to be temporarily increased by adopting strategies such as coating the probes with dissolvable materials^[^ ^11^ ^]^ or integrating them with a rigid shuttle device.^[^ ^12^ ^]^ Ironically, such strategies for insertion into the brain cause brain tissue damage due to the strong friction that occurs at the probe‐tissue interface owing to the probe's temporarily increased stiffness. Brain damage during insertion into the brain is also an unavoidable issue for neural probes with drug‐eluting coatings due to probe rigidity.^[^ ^13^ ^]^ Although anti‐inflammatory drugs eluted from the neural probe coatings suppress the increase in inflammation due to the chronic foreign body reaction, neuronal damage inflicted during the probe insertion still inevitably occurs. Moreover, an acute inflammatory response is inevitable as these drug‐eluting coatings are incapable of preventing the adsorption of proteins and debris from the punctured cellular membranes that initiate the inflammation signal cascades. In addition, the controlled release of the drugs from the coatings has proved to be challenging, and their limited drug loading capacity renders them unfeasible for long‐term usage.^[^ ^14^ ^]^

Another strategy to overcome gliosis caused by neural probes is the use of anti‐biofouling coatings to prevent undesired protein and cellular adhesion to the probe substrate, thus fundamentally eliminating the formation of scar tissue around the neural probe. Various hydrogels, such as polyethylene glycol, hyaluronic acid, and poly(oligoethylene glycol methacrylate) have received much attention as anti‐biofouling coating materials due to their nonadhesive properties resulting in the prevention of fouling and gliosis in vivo.^[^ ^15^ ^]^ However, the number of hydrogel‐based anti‐biofouling coatings is currently limited. Moreover, the hydrogel coating can increase the distance between the neurons and the electrodes by swelling and are also susceptible to delamination. These limitations make current hydrogel‐based anti‐biofouling coatings unfeasible for prolonged use on neural probes.

Recently, an approach to form an anti‐biofouling thin lubricant layer coating was developed based on inspiration from the *Nepenthes* pitcher plant.^[^ ^16^ ^]^ This lubricant‐infused slippery surface exhibits near‐frictionless properties, long‐term stability under liquid pressure conditions, and extreme liquid repellency against both nonpolar and polar liquids. Therefore, it has been studied for use as an anti‐biofouling coating for medical devices.^[^ ^17^ ^]^ However, no studies on whether this coating can be applied to electronic devices without compromising their signal recording performance and how it interacts in vivo have been conducted.

Herein, we report a lubricated immune‐stealthy probe surface (LIPS) fully combined with a probe that displays near‐frictionless and anti‐biofouling properties, thus maximizing its electrode performance in vivo. LIPS can repel various body fluids (e.g., artificial cerebrospinal fluid: aCSF; simulated body fluid: SBF; and blood) and maintain its extraordinary anti‐biofouling properties. Thus, this simple coating technique extends the lifetime of implantable devices by minimizing both the inflammatory response from probe insertion trauma and the adhesion of inflammatory factors. Its anti‐biofouling properties against plasma proteins and glial cells, resulting in less than 2% attachment, were confirmed in vitro. Moreover, LIPS reduced the impulse from probe‐tissue interface friction through mechanical analysis by 86%. We confirmed the enhanced electrophysiological performance of both short‐ and long‐term recording in the LIPS‐coated probe based on these advantages. Compared to the bare probe, the LIPS‐coated probe transiently doubled the number of recording channels and displayed up to a ninefold improvement in the signal‐to‐noise ratio (SNR) by minimizing insertion friction. The measurable signal period was also extended by twice as much (from 8 to 16 weeks) by minimizing the foreign body reaction around the probe. Therefore, we successfully demonstrated the potential feasibility of LIPS as a nonimmunogenic surface in vivo. We expect that LIPS will provide a new opportunity for the actual use of implanted devices in clinical BMI applications.

## 2 Results

### 2.1 LIPS for Neural Probes

**Figure 1:**
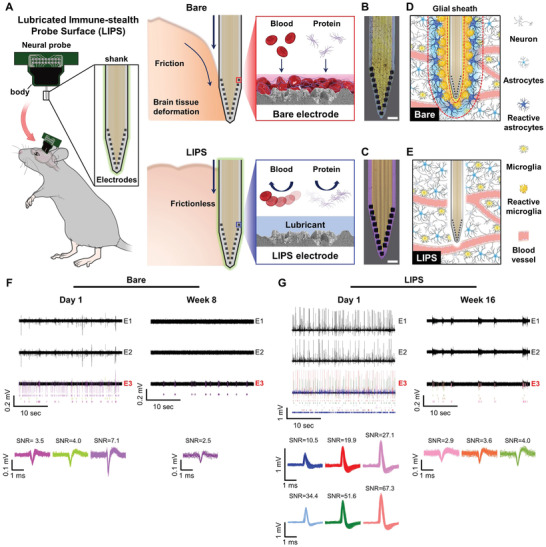
Lubricated immune‐stealthy probe surface (LIPS) for nonimmunogenic neural probes. A) Schematic of the mechanism for minimizing the immune response of the LIPS‐coated probe compared to the bare probe. Optical microscope images of the B) bare and C) LIPS probes submerged in blood for 30 min and retrieved (scale bars, 50 µm). Schematic of glial encapsulation on the D) bare and E) LIPS probes. F) Representative transient plot and sorted neural signals from the E3 of the bare probe on day 1 and week 8. G) Representative transient plot and sorted neural signals from the E3 of the LIPS‐coated probe on day 1 and week 16.

**Figure** **1A** shows a schematic of the insertion of bare and LIPS silicon‐based neural probes into mice. The bare probe coated with Pt black demonstrated decreased recording performance as insertion friction causes tissue damage and blood and protein adhere to electrode, but the LIPS‐coated probe demonstrated extended longevity and stability of sensing performance compared to the bare probe thanks to its near‐frictionless and anti‐biofouling properties obtained by exploring the biomimetic approach of mimicking the *Nepenthes* pitcher plant's frictionless surface. LIPS showed anti‐biofouling properties and long‐term stability by minimizing the adhesion of biological substances and the chemical affinity between the lubricant and the probe's surface. We confirmed LIPS' anti‐biofouling properties in vitro through optical microscopy imaging of bare and LIPS‐coated probes that had initially been immersed in blood. From these images, we observed adhesion of tissue debris on the bare probe mainly concentrated on the electrodes (Figure 1B). On the other hand, LIPS showed neither tissue debris adhesion (Figure 1C). Its anti‐biofouling properties acted as a barrier to glial cells whereas the bare probe was prone to their adhesion. Figure 1D,E shows schematics that explain the extent of glial encapsulation on the bare and LIPS‐coated probes. LIPS not only displayed anti‐biofouling properties but also minimized the acute insertion trauma, which is attributed to the near‐frictionless properties of the lubricant layer. Probe insertion friction does not simply cause an increase in acute neural loss, it also results in widespread disruption of the blood‐brain barrier (BBB).^[^ ^18^ ^]^ This leads to an influx of blood‐serum proteins (including albumin), thus activating inflammatory pathways in the nearby glial cells.^[^ ^19^ ^]^ Activated glial cells promote the initial formation of a glial sheath monolayer around the implanted probe.^[^ ^20^ ^]^ The glial sheath's thickness increases gradually due to chronic foreign body reaction, thereby driving away neurons from the electrodes and isolating the neural probe from the surrounding brain tissue.^[^ ^21^ ^]^ Since the recording of neural signals requires proximity between electrodes and neurons (<20 µm), the increased distance between them leads to a loss of reliable signaling.^[^ ^22^ ^]^ Figure 1F,G exhibits that there were more signals from the LIPS‐coated probe in both the short‐term and long‐term recordings. The SNR was also much greater for the LIPS‐coated probe than the bare probe throughout the entire signal collection period. Moreover, the LIPS‐coated probe demonstrated signal recordings for up to 16 weeks, which was twice that of the bare probe (8 weeks). These results suggest that the near‐frictionless and anti‐biofouling properties of LIPS have the capability of increasing not only the short‐term signal recording performance but also the life of the device.

### 2.2 Design, Fabrication, and Characterization of the LIPS‐Coated Probe

**Figure 2:**
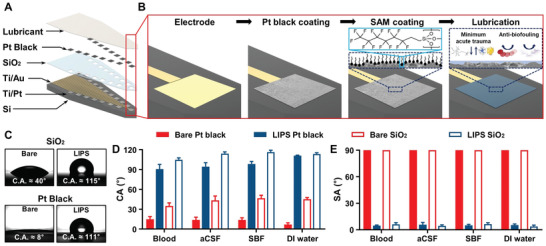
Design, fabrication process, and characterization of LIPS. A) Top‐to‐bottom exploded view of LIPS applied to a neural probe. B) Schematic of the LIPS fabrication process. The inset shows the self‐assembled monolayer formed on the substrate and nonimmunogenic mechanism due to the presence of the lubricant layer. C) Representative images of water droplets on bare SiO_2_, LIPS SiO_2_, bare Pt black, and LIPS Pt black. D) Contact angle (CA) and E) sliding angle (SA) measurements of various body fluids and deionized (DI) water on the different substrates. Data are obtained from distinct measurements on three different samples and at least at three different locations on each and presented as mean ± standard deviation (*n* = 9)

We designed a lubricated silicon‐based neural probe integrated with micro/nanostructured Pt black electrodes to bestow near‐frictionless and anti‐biofouling properties on the neural probes without compromising their electrode performance. **Figure** **2A** illustrates a top‐to‐bottom exploded view of LIPS applied to a neural probe consisting of 16 recording electrodes (each with an area of 14 × 14 µm^2^), a SiO_2_ layer and micro/nanostructured Pt black that had been chemically modified, and a lubricant layer. Figure 2B shows the simple fabrication process of the LIPS‐coated probe. First, a probe with Pt electrodes was prepared (Figure S1, Supporting Information). Second, Pt black layer was electroplated onto the Pt electrodes to compensate the increase in the impedance of the electrodes after coating lubricant layer by increasing the effective surface area of the electrodes. Scanning electron microscopy (SEM) and atomic force microscopy (AFM) images show the successful deposition of Pt black on the electrodes and the increased surface roughness (*R*_q_ = 0.35 µm) of the electrode surface (Figure S2, Supporting Information). Third, the probe's surface was functionalized with hydroxyl (–OH) groups via O_2_ plasma treatment, followed by the formation of a self‐assembled monolayer (SAM) with trichloro(1*H*,1*H*,2*H*,2*H*‐perfluorooctyl)‐silane (FOTS) using vapor‐phase deposition. Specifically, O_2_ plasma‐treated probes were first placed in a vacuum chamber and injected with FOTS vapor to conjugate the Si–Cl groups of the silanes with hydroxyl groups on the probe's surface. Afterward, deionized (DI) water vapor was injected to eliminate any unreacted Si–Cl groups. Finally, the chamber was pumped down to remove water vapor and vapor‐phase silane, thereby inducing condensation only in between the silanes bound to the surface. The chemical composition of the substrate surface was investigated after each round of coating using X‐ray photon spectroscopy (XPS) to confirm changes in the surface's chemical properties after O_2_ plasma treatment and FOTS deposition. Peaks of 532 eV in the O 1s region were observed in both the O_2_ plasma‐treated Pt black and SiO_2_ (Figure S3, Supporting Information), which can be attributed to Pt─OH and Si─OH bonds. After FOTS vapor‐phase deposition, there was a significant increase in the signal intensity of the CF_2_ and CF_3_ (689 eV) peaks in the F 1s region, suggesting that the fluorocarbon‐based silanes were successfully formed on both the Pt black surface and the SiO_2_ surface.

We then optimized the number of cycles for thin SAM formation to minimize the impedance increase of the electrodes by SAM deposition. We measured the liquid contact angle (CA) and impedance measurements to confirm the changes in electrode surface energy and electrode performance during each cycle. Both CA and impedance increased in the first and second cycles, but only impedance rose consistently in the following cycles (Figure S4, Supporting Information). Therefore, the number of SAM‐coating cycles required to lower the electrode's surface energy to increase chemical affinity between the surface and slippery lubricant while minimizing the impedance increase was optimized to two. Finally, the SAM‐coated probe was lubricated by dipping it into the perfluoropolyether lubricant, after which we measured the electrodes' electrical impedance to evaluate the electrode performance after lubrication. At 1 kHz, the measured impedances of the LIPS‐coated probe and the bare probe without Pt black were 613 ± 41 and 986 ± 55 kΩ, respectively (Figure S5, Supporting Information). Thus, we confirmed that the impedance decrease by Pt black was more than enough to compensate the impedance increase caused by the lubricant layer on the SAM coating, ultimately resulting in lower the impedance than that of the Pt electrode surface (Figure S5, Supporting Information). In conclusion, SAM coating and lubrication of the neural probe did increase its impedance from that of Pt black coated bare probe, but the measured impedance of LIPS was sufficiently low to record neural signals from individual neurons^[^ ^23^ ^]^ and even lower than that of bare electrode without Pt black.

We confirmed the CAs of the SiO_2_ and Pt black surfaces (the top surface materials of the neural probe) before and after LIPS coating. Figure 2C shows representative CA images of DI water droplets on the SiO_2_ and Pt black surfaces before and after LIPS coating. The CA images indicate that bare SiO_2_ and Pt black are both hydrophilic; this is especially evident in the image of Pt black, which displays complete wetting of the surface. Its rough surface by electroplating led to enhancement of its hydrophilicity, ultimately making it superhydrophilic.^[^ ^24^ ^]^ On the other hand, LIPS Pt black and SiO_2_ both led to a CA of around 110° due to the lubricant layer. Finally, to demonstrate the liquid repellency and nonadhesive properties of LIPS, the CAs and sliding angles (SAs) of various bodily fluids (aCSF, SBF, and blood) and DI water were measured on both LIPS‐treated and bare substrates (Figure 2D,E). The liquids on the bare substrates demonstrated comparatively low CAs and were pinned to the substrates without sliding off. On the LIPS‐treated substrates, all of the liquids showed CAs of ≈110° regardless of the substrate material, and remarkably low SAs were observed, thereby causing the liquids to roll off when tilted at an angle of below 8°. These results are strongly indicative of the nonadhesive properties of LIPS.

### 2.3 The Near‐Frictionless Property of LIPS‐Coated Probes

**Figure 3:**
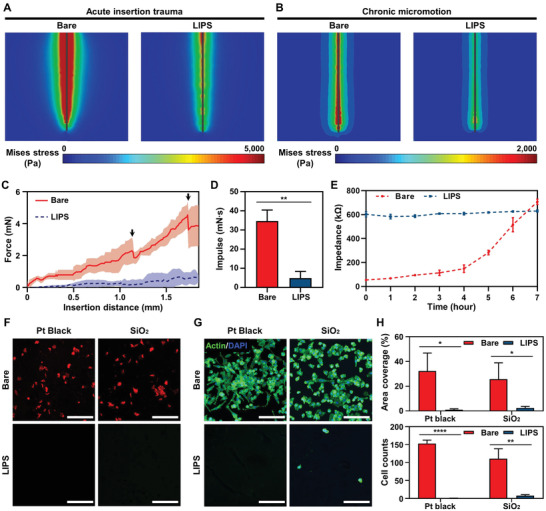
Near‐frictionless and anti‐biofouling properties of LIPS in the presence of blood, protein, and cells. A) von Mises stress profiles of bare and LIPS‐coated probes within brain tissue during insertion. B) von Mises stress profiles of the bare and LIPS‐coated probes within brain tissue under lateral micromotion of 100 µm. C) Force measurements during insertion of the bare and LIPS‐coated probes at 0.1 mm s^−1^ into the brain phantom. D) Applied impulse on the brain phantom during insertion of the bare and LIPS‐coated probes (^**^ *p* = 0.0015, *n* = 3: *n* is the number of the repeated measurements). E) Plots of impedance change over time of the probes immersed in blood. F) Confocal fluorescence microscopy images of protein (red; albumin) on bare and LIPS‐coated SiO_2_ and Pt black (scale bars, 400 µm). G) Confocal fluorescence microscopy images of the cells (microglia) adhered to bare and LIPS‐coated SiO_2_ and Pt black (scale bars, 400 µm). H) Calculated area coverage and cell counts of inflammatory protein, albumin, and the number of microglia adhered to each substrate (area coverage: ^*^ *p* = 0.0208, *n* = 3 for Pt black; ^*^ *p* = 0.0398, *n* = 3 for SiO_2_; cell counts: ^****^ *p* < 0.0001, *n* = 3 for Pt black; ^**^ *p* = 0.0031, *n* = 3 for SiO_2_: *n* is the number of samples). Data are presented as mean ± standard deviation. Statistical significance was tested with two‐tailed unpaired *t*‐tests. ^*^ *p* < 0.05; ^**^ *p* < 0.01; ^***^ *p* < 0.001; ^****^ *p* < 0.0001; NS: no significant difference.

The most predominant cause of acute inflammation is insertion trauma.^[^ ^25^ ^]^ Intense acute insertion trauma can be ensued by neural loss around the insertion site and can accelerate chronic inflammation. Furthermore, a major cause of chronic inflammation is brain micromotion and subsequent foreign body reaction.^[^ ^26^ ^]^ Since acute insertion trauma and micromotion are affected by friction at the probe‐tissue interface, it is very important to minimize it. We used a finite element analysis model that mimicked the experimental and physiological conditions for probe insertion and brain micromotion to predict the mechanical interaction between the LIPS‐coated probe and the brain tissue in silico.^[^ ^27^ ^]^ First, to evaluate the effect of probe insertion on brain tissue, we observed the distribution of von Mises stress by inserting bare and LIPS‐coated probes to a depth of 1.85 mm into a cube with the elastic modulus (*E* = 500 Pa) of brain tissue. Thus, we confirmed that von Mises stress was concentrated on the surface of both inserted probe types. Still, the surrounding tissue area was influenced by von Mises stress and its intensity level was distinctly lower for the LIPS‐coated probe than the bare probe (**Figure** **3A**). In addition, to evaluate the effect of probe micromotion on brain tissue, the probes were inserted to a depth of 1.85 mm into the cube, and the cube was moved back and forth three times laterally by 100 µm. The results show that von Mises stress is concentrated at the tips of both the LIPS and bare probes, but greater stress occurred over a wider range around the bare probe (Figure 3B). In conclusion, the reduced stress at the end of the LIPS‐coated probe suggests that the chronic immune response around the electrodes can be reduced since they are near the tip of the probe.

To validate the simulation results, we performed insertion tests in brain tissue phantoms. In the test, we used artificial brain phantoms implemented with 0.6% agarose gel, which is known to have very similar mechanical properties to the brain^[^ ^28^ ^]^ (Figure S6, Supporting Information). Friction between the probe and the brain phantom was measured by placing the brain‐shaped agarose gel on a force sensor and inserting the probe at a constant speed (0.1 mm s^−1^). For both probe types, the friction force between the probe and the brain phantom increased almost linearly with probe insertion distance until the advancement of the probe stopped, which can be attributed to the increasing area of contact between the probe and the brain phantom (Figure 3C). However, a big difference in the maximum friction force during both bare probe and LIPS‐coated probe insertion was observed due to the lubricant layer of the LIPS‐coated probe ensuring its near‐frictionless property. The maximum friction force of the bare probe was 4.7 mN, which was eight times the maximum friction force of the LIPS‐coated probe (0.56 mN). Moreover, there was often a sudden drop in friction force when inserting the bare probe, which could have been due to the accumulation of gel fragments at the needle and facilitated by the hydrophilicity of the bare probe's surface.^[^ ^29^ ^]^ Hence, release/penetration of these accumulated materials could have resulted in the rapid decline in friction force. Notably, there was no rapid drop in friction force when the LIPS‐coated probe was inserted, which is attributed to its anti‐adhesion properties. In addition, we calculated the tissue damage caused by the friction between the probe and the brain based on measured friction force from the force sensor. After calculation of the friction (*F*_friction_) generated during probe insertion as a function of time (*S*), tissue damage (*J*_damage_) caused by this friction can be quantified using the formula below^[^ ^30^ ^]^

(1) $$J_{\mathrm{damage}}=\;\int_{0}^{s}F_{\mathrm{friction}}\;dt$$

Based on this formula, we calculated the impulse applied to the brain tissue during insertion. An impulse of 34.6 ± 3.5 mN∙s was applied to the brain tissue phantom during the insertion of the bare probe whereas only 4.9 ± 3.1 mN∙s was applied to the LIPS‐coated probe due to its near‐frictionless property (Figure 3D). These results show that the amount of impact applied to brain tissue during probe insertion is reduced by 86% using LIPS, which means that acute inflammation caused by insertion trauma can be drastically reduced. Finally, to evaluate the degradation of LIPS caused by mechanical stress, we repeatedly measured the LIPS‐coated probe's electrical impedance after each cycle of probe insertion and extraction from the brain phantom. A slight decrease in impedance was observed in the first cycle caused by excess lubricant removal, but the impedance remained constant in subsequent cycles (Figure S7, Supporting Information). Based on this result, we can confirm the sufficient mechanical durability of the LIPS‐coated probe against the mechanical stress applied during insertion.

### 2.4 The Anti‐Biofouling Property of the LIPS‐Coated Probe

During probe insertion, the BBB is damaged as the probe penetrates the neurovasculature of the brain, thereby inducing the release of blood plasma into the surrounding tissue.^[^ ^19^ ^]^ This acute insertion trauma causes inflammatory factors from the destroyed BBB to gather around the inserted probe, which facilitates the activation of astrocytes and microglia.^[^ ^31^ ^]^ As such, blood clots and plasma proteins adsorbed to the probe's surface can sustain the release of pro‐inflammatory cytokines, thereby recruiting macrophages, activating their differentiation, and promoting gliosis progression.^[^ ^32^ ^]^ Therefore, it is essential to prevent surface adsorption and aggregation of blood and proteins on the probe's surface during the early stages of gliosis. To confirm the anti‐biofouling performance of the LIPS‐coated probe, it was incubated in a suspension of blood and albumin, which is a blood plasma protein responsible for inducing glial cell activation and proliferation.^[^ ^33^ ^]^ During the incubation period, impedance similar to the initial value was recorded (Figure 3E). In contrast, the impedance of the bare probe gradually increased and exceeded the LIPS‐coated probe's impedance by around 70 kΩ after 7 h. Subsequently, the anti‐biofouling properties of LIPS on SiO_2_ and Pt black substrates were tested against inflammatory protein and cell representatives albumin and microglia, respectively. As shown in the fluorescence microscopy images, many proteins were observed on the Pt black and bare SiO_2_ substrates incubated in the albumin suspension (Figure 3F). However, there were hardly any proteins present on the LIPS substrate due to its excellent anti‐biofouling properties. Moreover, microglia were cultured on the substrates to produce an in vitro model of glial scarring (Figure 3G). After 48 h, cells had adhered over nearly all of the bare Pt black and bare SiO_2_ surfaces. The cells on bare Pt black especially showed increased adhesion and proliferation while displaying multiple morphologies, such as polygonal, bipolar, and spherical shapes. This is attributed to the increased surface area of the Pt black substrate enabling more protein adsorption and cell adhesion.^[^ ^34^ ^]^ On the other hand, LIPS maintained its anti‐biofouling properties, as confirmed by the absence of microglial cell adhesion. Thus, we confirmed that LIPS exhibited anti‐biofouling properties against all biological substances compared to the bare probe's surface materials through fluorescence intensity‐based quantification of protein adhesion and cell counting: LIPS exhibited 98.3% less adhesion compared to the bare surface (Figure 3H). From the in vitro experiment results, we found that the application of LIPS to neural probes not only minimized acute tissue damage caused by friction between the probe and its surrounding tissue but also reduced chronic tissue response by preventing surface adhesion of inflammatory factors.

### 2.5 Short‐Term Neural Recording Quality Comparison of Bare and LIPS‐Coated Probes

**Figure 4:**
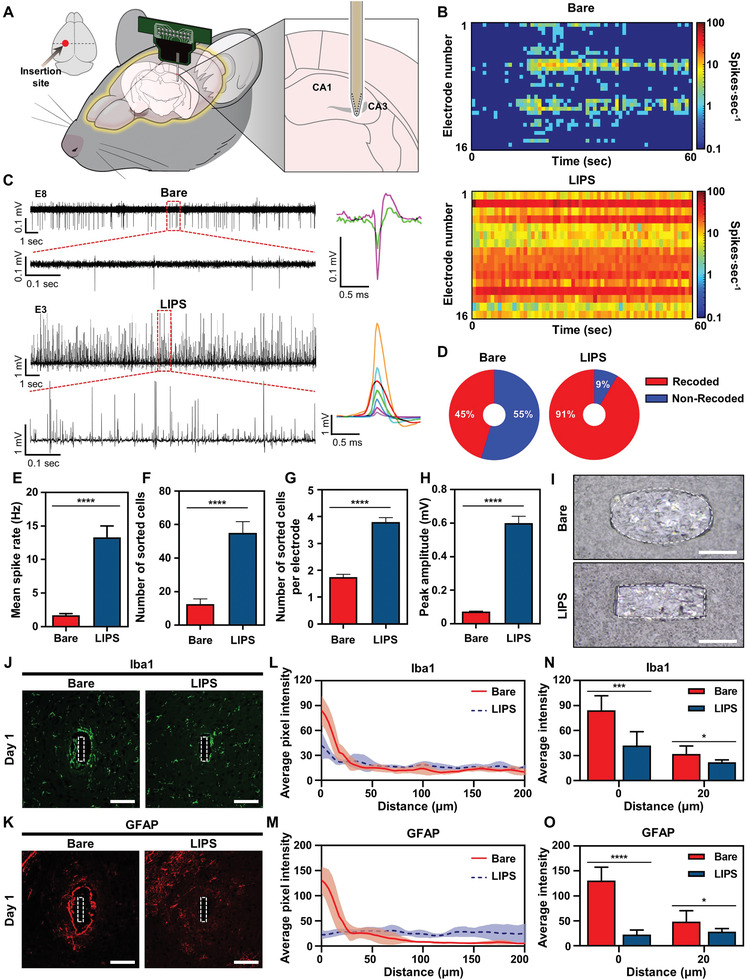
Comparison of short‐term neural recording quality and immune responses of coated and uncoated probes on day 1. A) Schematic diagram showing the probe inserted into the hippocampal CA3 region of the mouse brain. B) Color‐mapped raster plot showing neural activities recorded by the bare and LIPS‐coated probes. C) Neural signals recorded from the electrodes in the bare and LIPS‐coated probes. D) Pie charts showing the percentage of the signal‐recorded and signal‐not‐detected electrodes in the bare and LIPS‐coated probes. E) Comparison of the mean spike rate of the measured neural signals between the bare and LIPS‐coated probes (^****^ *p* < 0.0001, *n* = 58 and 116 for the bare and LIPS‐coated probes, respectively: *n* is the number of signal‐recorded electrodes). F) Comparison of the number of measured neural cells from the bare and LIPS‐coated probes (^****^ *p* < 0.0001, *n* = 8 for both: *n* is the number of recorded mice). G) Comparison of the measured neural cells per electrode between the bare and LIPS‐coated probes (^****^ *p* < 0.0001, *n* = 58 and 116 for the bare and LIPS‐coated probes, respectively: *n* is the number of signal‐recorded electrodes). H) Comparison of the peak amplitudes of the measured neural signals between the bare and LIPS‐coated probes (^****^ *p* < 0.0001, *n* = 100 and 440 for the bare and LIPS‐coated probes, respectively: *n* is the number of sorted neural signals). I) Optical images showing the size of the insertion damage area in brain slices after insertion of the bare and LIPS‐coated probes (scale bars, 50 µm). Confocal fluorescence microscopy images tagged for J) Iba1 and K) GFAP in the tissue surrounding the bare or LIPS‐coated probe proximity (scale bars, 100 µm). The quantified expression of L) Iba1 and M) GFAP as a function of distance from the probe‐tissue interface (line, average intensity; shaded area, standard error of the mean). N) The average Iba1 expression intensity from 0 and 20 µm away from the probe insertion site (^***^ *p* = 0.0006, *n* = 7 at 0 µm; ^*^ *p* = 0.0222, *n* = 7 at 20 µm: *n* is the number of analyzed images). O) The average GFAP expression intensity from 0 and 20 µm away from the probe insertion site (^****^ *p* < 0.0001, *n* = 10 at 0 µm; ^*^ *p* = 0.0111, *n* = 10 at 20 µm: *n* is the number of analyzed images). Data in (E–H) are presented as mean ± standard error of the mean. Data in (N–O) are presented as mean ± standard deviation. Statistical significance was tested with two‐tailed unpaired *t*‐tests. ^*^ *p* < 0.05; ^**^ *p* < 0.01; ^***^ *p* < 0.001; ^****^ *p* < 0.0001; NS: no significant difference.

To confirm the improvement of short‐term neural recording quality due to insertion trauma lowered by the low friction force of LIPS, we measured neural activities from the hippocampal CA3 region of a mouse brain using bare and LIPS‐coated probes (**Figure** **4A**). Neural signals from eight mice per each type of probe were recorded for an accurate comparison of the short‐term neural recording quality between the two probes. Notably, neural signals were recorded from most of the electrodes on the LIPS‐coated probe (Figure 4B), while the overall firing rate of the measured signals at each electrode was significantly higher than that of the bare probe (Figure 4B). Furthermore, the amplitudes of the measured signals at the electrode of the LIPS‐coated probe were significantly larger than those of the bare probe, and positive‐first signals were observed at the electrode of the LIPS‐coated probe (Figure 4C). In general, for extracellular recordings, the closer the distance between the electrode and the neuron, the greater the amplitude of the neural signal.^[^ ^35^ ^]^ Moreover, a positive‐first signal is observed when the electrode is located close to the cell body of a neuron,^[^ ^36^, ^37^ ^]^ and large signals above 1 mV are measured when the cell body and electrode are in contact.^[^ ^36^ ^]^ Based on these facts, we conclude that there were cell bodies of neurons close to the electrodes of the LIPS‐coated probe and that some were even in contact with the electrode.

We performed a quantitative analysis of the neural signals measured from LIPS and bare probes. Neural signals from 116 of the 128 electrodes using the LIPS‐coated probe (91%) and 58 of the 128 electrodes using the bare probe (45%) were obtained (Figure 4D). Also, the frequency of the neural spikes measured by the two probes showed a significant difference (Figure 4E). Furthermore, we classified neural signals measured from LIPS and bare probes using PCA and k‐means clustering. The number of sorted neurons from the LIPS probe was about four times greater than that of the bare probe (Figure 4F), and the number of neurons measured from each electrode was about two times greater in the LIPS probe (Figure 4G). These results mean that neural signals from more neurons were recorded at the electrodes of the LIPS‐coated probe. Finally, most neural signals measured on the bare probe were found to be within 0.2 mV, while neural signals larger than 0.5 mV were usually observed on the LIPS‐coated probe (Figure 4H). Thus, there was also a significant difference in the magnitude of the measured neural signals. Based on these results, we can confirm that the LIPS‐coated probe is far superior to the bare probe in short‐term neural recording quality.

The sizes of the brain‐damage areas inflicted by insertion of the bare and LIPS‐coated probes were observed and compared to confirm the minimized brain tissue damage following the application of LIPS. Insertion of the LIPS‐coated probe resulted in a brain‐damage area that was similar to the size of the LIPS‐coated probe whereas insertion of the bare probe resulted in a brain‐damage area that was larger than the bare probe (Figure 4I). Considering that the measurable signal distance was estimated at around 20 µm between the electrode and neurons,^[^ ^38^ ^]^ we can conclude that improved short‐term neural recording quality of the LIPS‐coated probes resulted from the minimized brain tissue damage caused by insertion.

### 2.6 Acute Immune Response Assessment of Bare and LIPS‐Coated Probes

The results for the immune‐histochemical analysis of brain slices 1 day after implantation of the LIPS and bare probes were compared to assess the acute immune response to the surgery. The expression of ionized calcium‐binding adaptor molecule 1 (Iba1) and glial fibrillary acidic protein (GFAP) was used to assess activated microglia and astrocytes, respectively.^[^ ^39^ ^]^ Confocal fluorescence microscopy images showed significantly lower expression of Iba1 and GFAP around the insertion site of the LIPS‐coated probe and similar sizes between the damaged area and the LIPS‐coated probe (Figure 4J,K). In contrast, intense expression of Iba1 and GFAP was found around the insertion site of the bare probe, which showed the active progression of acute immune response by microglia and astrocytes (Figure 4J,K). Moreover, the damaged area due to insertion of the bare probe was 120% larger than the LIPS‐coated probe, which was due to the relatively strong friction at the probe‐tissue interface (Figure S8, Supporting Information). The acute immune response was quantified as a function of the distance from the probe‐tissue interface to quantitatively compare the responses of the two probes (Figure 4L–O). The fluorescence intensity values were calculated on 360 equidistant radial lines from the center of the implant void and averaged for each image. The LIPS‐coated probe yielded significantly less Iba1 expression by microglia than the bare probe at both the 0 and 20 µm distance points (Figure 4N). Microglia activation around the LIPS‐coated probe was caused by inevitable BBB damage during probe insertion.^[^ ^40^ ^]^ Unlike microglia, activated astrocytes showed a distinct difference in GFAP expression levels between the bare and LIPS‐coated probes (Figure 4J). Bare probes showed intense astrocyte activation compared to LIPS‐coated probes at both the 0 and 20 µm distance points (Figure 4O). In conclusion, we determined that the LIPS‐coated probe caused less damage during the brain insertion than the bare probe due to the reduced friction between the probe and the brain, thus the acute immune response was significantly suppressed.

### 2.7 Long‐Term Neural Recording Quality Comparison of the Bare and LIPS‐Coated Probes

**Figure 5:**
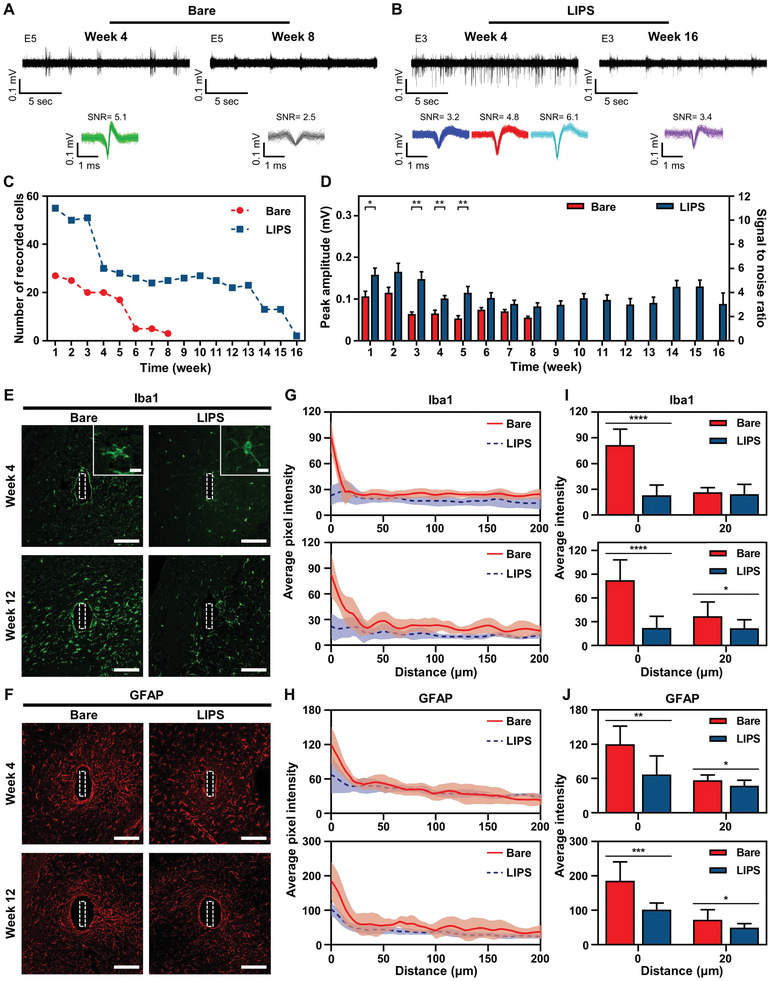
Comparison of long‐term neural recording quality and immune responses of coated and bare probes at 1 and 3 months. The representative neural signals recorded at A) the electrode of the bare probe at 4 and 8 weeks and B) the LIPS‐coated probe at 4 and 16 weeks. C) The number of recorded neural cells by the bare and LIPS‐coated probes over time. D) Peak amplitude and signal‐to‐noise ratio (SNR) of recorded neural cells by the bare and LIPS‐coated probes over time. (^*^ *p* = 0.0337, *n* = 27 and 55 for the bare and LIPS‐coated probes at 1 week, respectively; ^**^ *p* = 0.004, *n* = 20 and 50 for the bare and LIPS‐coated probes at 3 weeks, respectively; ^**^ *p* = 0.0013, *n* = 20 and 30 for the bare and LIPS‐coated probes at 4 weeks, respectively; ^**^ *p* = 0.0031, *n* = 17 and 28 for the bare and LIPS‐coated probes at 5 weeks, respectively; *n* is the number of sorted neural cells). Confocal fluorescence microscopy images tagged for E) Iba1 and F) GFAP in the tissue surrounding the bare or LIPS‐coated probes (scale bars, 100 µm). The inset images show differences in the microglia morphology between the bare and LIPS‐coated probe (scale bars, 10 µm). The quantified expression of G) Iba1 and H) GFAP as a function of distance from the probe‐tissue interface at 4 weeks (upper) and 12 weeks (lower) (line, average intensity; shaded area, standard error of the mean). I) The average Iba1 expression intensity at 4 weeks (upper) and 12 weeks (lower) from 0 and 20 µm away from the probe insertion site (4 weeks: ^****^ *p* < 0.0001, *n* = 10 at 0 µm; 12 weeks: ^****^ *p* < 0.0001, *n* = 10 at 0 µm; ^*^ *p* = 0.0323, *n* = 10 at 20 µm: *n* is the number of analyzed images). J) The average GFAP expression intensity at 4 weeks (upper) and 12 weeks (lower) from 0 and 20 µm away from the probe insertion site (4 weeks: ^**^ *p* = 0.0018, *n* = 10 at 0 µm; ^*^ *p* = 0.0332, *n* = 10 at 20 µm; 12 weeks: ^***^ *p* = 0.0003, *n* = 10 at 0 µm; ^*^ *p* = 0.0339, *n* = 10 at 20 µm: *n* is the number of analyzed images). Data in (D) are presented as mean ± standard error of the mean. Data in (I,J) are presented as mean ± standard deviation. Statistical significance was tested with two‐tailed unpaired *t*‐tests. ^*^ *p* < 0.05; ^**^ *p* < 0.01; ^***^ *p* < 0.001; ^****^ *p* < 0.0001; NS: no significant difference.

We measured neural signals from three mice using bare and LIPS‐coated probes weekly to confirm the improvement in long‐term neural recording quality resulting from 1) the prevention of biofouling and 2) the reduction of micromotion due to the near‐frictionless lubricant layer of LIPS. Neural signals were measured from mice anesthetized with 1% isoflurane to minimize the electrical artifacts due to movement by the mice. The amplitudes of neural signals measured from the bare probe were significantly decreased at week 8 compared to week 4 (**Figure** **5A**). On the other hand, although some of the neural signals disappeared in the LIPS‐coated probe, a signal with a shape and size similar to that observed at week 4 was observed at week 16 (Figure 5B). We analyzed the number and amplitudes of the neural signals measured to quantitatively compare the long‐term neural recording quality of the two probes. We confirmed that the number of neural signals measured by the LIPS‐coated probes in the first week was more than twice the number of those measured by the bare probe (Figure 5C). In addition, the amplitudes of the neural signals and SNR were around 1.5 times higher for the LIPS‐coated probes in the first week (Figure 5D). The number and amplitude of neural signals measured by the bare probe decreased over time but were detectable for up to 8 weeks (Figure 5C,D). Similarly, the number of neural signals measured by the LIPS‐coated probe gradually decreased over time but was detectable up to 16 weeks, which is twice that of the bare probe (Figure 5C). Moreover, although the amplitude of the neural signals measured from the LIPS‐coated probe showed some deviation up to 16 weeks micromotion in the brain, it was stable overall (Figure 5D). Hence, we can confirm that LIPS significantly improved the long‐term neural recording quality of the neural probe.

### 2.8 Chronic Immune Response Assessment of Bare and LIPS‐Coated Probes

Results of the immunohistochemical analysis of brain slices were compared 4 and 12 weeks after implantation to assess the chronic immune response to the LIPS and bare probes. Confocal fluorescence microscopy images showed significantly lower expression of Iba1 around the insertion site of the LIPS‐coated probe at both 4 and 12 weeks after implantation (Figure 5E). Notably, the images also show a remarkable difference in the morphology of microglia at 4 weeks. A number of activated microglia with amoeboid shapes were observed around the bare probe insertion site, while only ramified microglia were observed around the LIPS‐coated probe insertion site. This result suggests that microglial activation, proliferation, migration, and glial encapsulation actively progressed in the bare probe.^[^ ^41^ ^]^ Thus, we determined that the near‐frictionless and anti‐biofouling properties of LIPS can prevent the aggregation and activation of microglia in the long‐term. Moreover, the expression of GFAP was found to be significantly lower around the insertion site of the LIPS‐coated probe (Figure 5F). We quantified the expressions of Iba1 and GFAP as a function of the distance from the probe‐tissue interface to quantitatively compare the chronic immune response between two probes (Figure 5G,H). The bare probe showed intense expression of Iba1 both 4 and 12 weeks after probe implantation, whereas the LIPS‐coated probe displayed significantly lower expression of Iba1 (Figure 5G). Moreover, both the bare and LIPS‐coated probes showed local expression of GFAP at the probe‐tissue interface that gradually decreased with increasing distance (Figure 5H), although there were differences in the degree of expression. The LIPS‐coated probe yielded significantly lower numbers of microglia at distances of 0 and 20 µm from the probe‐tissue interface (Figure 5I). In addition, LIPS showed significantly low expression of GFAP at both distances (Figure 5J). Based on these results, we can deduce that the drastic neural recording deterioration in the bare probe was due to microglia and astrocyte activation and conclude that the neural signals were available for longer in the LIPS‐coated probe due to the relatively low immune response and less brain tissue damage. Overall, in terms of short‐ and long‐term recording, we ascertained that LIPS maximizes the longevity and recording performance of silicon‐based neural probes by preventing biofouling and reducing brain damage during insertion and the mechanical mismatch between the neural probe and the brain tissue.

## 3 Discussion

LIPS provides a novel strategy for improving both short‐ and long‐term signal recording by neural probes. LIPS allows near‐frictionless probe insertion and bestows anti‐biofouling properties that can minimize acute inflammation from insertion trauma and chronic inflammation resulting from micromotion and adhesion of inflammatory cells. Various characterization methods, including SEM, AFM, XPS, CA measurements, and electrochemical impedance spectroscopy (EIS) were utilized to optimize the fabrication conditions for LIPS without compromising the signal recording performance. LIPS was then tested in vitro to demonstrate its near‐frictionless coating of a probe by using a brain phantom. The results show that the maximum friction force during probe insertion was eight times less than with a bare probe, thus the calculated impulse applied to brain tissue phantom was reduced by 86%, which shortened the acute inflammatory response that followed. Moreover, the anti‐biofouling properties of LIPS against body fluids, proteins, and glia cells were tested. Due to its nonadhesive, LIPS showed stable and excellent anti‐biofouling properties in a biological environment. Using these properties, we further investigated the nonimmunogenicity and electrophysiological performance of the LIPS‐coated probe in vivo. During short‐term recording, the LIPS‐coated probe showed a 102% increase in the number of signal recording channels and a significant increase in the number and amplitude of measured signals per electrode, without cell death around the probe by biocompatibility of LIPS.^[^ ^17b^ ^]^ Moreover, in long‐term recording, the LIPS‐coated probe recorded more neural signals over a period twofold longer than the bare probe. The immune response around the insertion site was assessed using immunohistochemistry at 1 day, 4 weeks, and 12 weeks. It showed significantly lower expression levels of Iba1 and GFAP in microglia and astrocytes within close proximity to the LIPS‐coated probe compared to the bare probe for all periods. To summarize, we demonstrated that LIPS provides near‐frictionless and anti‐biofouling properties, thus significantly minimize gliosis around the probe, which interferes with measuring neural signals by the electrode.^[^ ^8b^ ^]^ Therefore, LIPS could improve the recording quality of a neural probe in both the short‐term and long‐term applications.

Our surface modification technique has several advantages over previous techniques for long‐term neural recording by minimizing the immune response. First, both long‐term and short‐term signal quality were significantly improved. Current flexible probes have difficulty measuring neural signals immediately after insertion due to acute brain damage caused by insertion^[^ ^12a,c,d^ ^]^ whereas our technique minimized acute brain damage by reducing the friction between the brain and the probe. Thus, large neural signals of 1 mV or more from the electrodes were recorded. Improvement in the short‐term recording quality is a highly critical consideration in terms of neuroscience given that most neural circuit studies have been carried out on anesthetized animals. Second, our surface modification technique can be applied to any implantable device, including silicon‐based neural probes. Considering that the devices used in neuroscience such as silicon‐based neural probes and tetrodes are commercially available, our technology can provide higher versatility and accessibility than previous non‐commercial devices such as flexible probes. Despite the enhanced neural signal recording performance in both the short‐term and long‐term of LIPS, the increase of impedance caused by the lubricant coating on the electrode surface and stability in dynamic physiological environments still remains a challenge. Further research is required to develop advanced surface modification techniques that can selectively coat LIPS while leaving out electrode surfaces to prevent the impedance's increase of the electrode. Also, investigations on advanced coating materials, which can effectively hold lubricant, are demanded to maintain the immunogenic properties even in long‐term chronic applications.

In conclusion, LIPS not only addresses existing challenges of commercial implantable devices such as silicon‐based neural probes and deep brain stimulators (DBSs) but also offers a promising biocompatible coating for any implantable medical device. Notably, LIPS can maximize the recording performance and the longevity of implanted devices used for BMI. Overall, LIPS is a promising nonimmunogenic coating technique that provides minimal acute insertion trauma and excellent anti‐biofouling properties that reduce gliosis. We expect that our LIPS technology will serve as a catalyst for the practical application of BMI.

## 4 Experimental Section

### Fabrication of the Neural Probe with Pt Black Electrodes

The silicon‐based microelectromechanical systems (MEMS) neural probe was fabricated by using previously developed processes.^[^ ^42^ ^]^ First, a 400 nm thick SiO_2_ layer for electrical insulation was deposited through plasma‐enhanced chemical vapor deposition on a silicon‐on‐oxide (SOI) wafer with a 40 µm thick top silicon layer (Figure S1, Supporting Information). After consecutive Ti/Au (200 Å/3000 Å) deposition and patterning of the signal line, a second 400 nm thick SiO_2_ layer was deposited on top of the silicon layer of the SOI wafer (Figure S1, Supporting Information). After patterning the electrode sites, the insulation layer on the electrode sites was etched using reactive ion etching (RIE), and Ti/Pt (200 Å/1500 Å) was deposited on the electrode sites through a lift‐off process. Finally, one shank of the neural probe was released through consecutive deep RIE and RIE.

The fabricated neural probe was attached to a custom printed circuit board (PCB) through a fast adhesive (Loctite 401, LOCTITE, Germany) and wire‐bonded between the electrical pads of the neural probe and Au‐exposed pads of the PCB using a wire‐bonder (Kulicke and Soffa model 4526, Kulicke and Soffa, Republic of Singapore). Finally, the wire‐bonded area was protected with a thermal epoxy (EPO‐TEK 320, Epoxy Technology, Inc., USA) and an FPC connector (503480‐1000, Molex, USA) was attached to the PCB.

Pt black was electroplated on the Pt electrodes of the neural probe to improve the quality of the neural signal recording. Pt black electroplating solution was prepared by mixing hexachloroplatinic acid hydrate (HCPA), 0.025 n HCl, and 0.025% lead acetate in deionized water.^[^ ^43^ ^]^ Next, the neural probe was immersed in the electroplating solution with Pt wire (the reference electrode) and Ag/AgCl wire (the counter electrode). Pt particles in the Pt black electroplating solution were electrically deposited on Pt electrodes by applying an electrical potential of 0.2 V for 35 s through a potentiostat (PalmSens3, PalmSens, Netherlands).

### Fabrication of the LIPS Neural Probe

The probe was plasma‐treated under ambient oxygen (100 W, 5 min) to form hydroxyl (–OH) terminal groups and then immediately relocated into a custom‐made multi‐valve vacuum chamber (1 Torr). Another pre‐degassed (1 Torr) vacuum chamber with a glass vial containing 200 µL of FOTS (Sigma‐Aldrich, Germany) was connected to the custom‐made multi‐valve chamber containing the probe and kept at 60 °C for 30 min. Afterward, the valve supplying FOTS vapor was closed and another chamber filled with vaporized DI water was connected to the multi‐valve chamber to supply water vapor. After 30 min, this chamber was isolated and degasified to 1 Torr, which was repeated twice to cover any pinholes in the SAM. Next, the resulting FOTS SAM‐formed probe was cleaned with isopropyl alcohol (IPA) and DI water to remove excess FOTS. After filtering the perfluoropolyether (PFPE) lubricant (Krytox 101, immiscible with most nonpolar and polar liquids; chemically stable; DuPont, USA) through a 0.2 µm filter, the probe was lubricated by dipping it into the lubricant. The excess lubricant was removed by tilting the probe at an angle of 90° for 24 h, after which the resulting probe was immediately used for surgery.

### Surface Characterization of LIPS

Surface morphologies of the substrates and neural probes were examined using field‐emission SEM (7610f‐plus, JEOL, Japan) and AFM (XE‐100, Park Systems, Korea). Surface roughness (*R*_a_, *R*_q_) was measured over an area of 10 × 10 µm^2^ (*n* = 3). Surface chemical composition was analyzed using energy‐dispersive X‐ray spectroscopy (EDS) equipped with SEM. Liquid CA and SA were measured using a CA measurement system equipped with a dynamic image capture camera (Smart Drop, FEMTOBIOMED, Korea). Tilted‐drop measurements were conducted to obtain SAs using 7 µL droplets of various liquids (aCSF, SBF, blood, and DI water). The tilted‐drop method begins by placing a droplet of each liquid on the samples and gently tilting at 1° s^−1^ until the droplet rolls off. The angle at which the droplet rolls off is the SA (*n* = 5).

### Numerical Analysis of Insertion Trauma and Brain Micromotion

First, 3D models of a cube (2 × 2 × 2 mm^3^) to be used as brain tissue and a real‐sized probe were produced through Fusion 360 (Autodesk, California, USA). Next, 3D finite element models were established by importing STEP files of the cube and probe into multi‐body dynamics software RecurDyn (FunctionBay, Inc., Korea). The physical properties of the cube were set to the same elasticity^[^ ^44^ ^]^ (*E* = 500 Pa) and damping coefficient^[^ ^45^ ^]^ (*ζ* = 0.25) as brain tissue and converted to a structured mesh grid. To simulate the effect of probe insertion on brain tissue, surface‐to‐surface contact model was established using the friction coefficient of 0.3705 (bare SiO_2_) and 0.0391 (lubricated SiO_2_). Then the probe was placed 1 mm above the center of the cube fixed to the ground and inserted to a depth of 1.85 mm at 0.1 mm s^−1^ in the vertical direction. The analysis was carried out in 3000 steps for 30 s, and the stress distribution was confirmed by observing the contours of von Mises stress within the cube. To simulate brain micromotion, the probe was inserted to a depth of 1.85 mm into the cube, and the cube was moved back and forth three times laterally by 100 µm. The analysis was performed in 600 steps for 60 s, and the stress distribution was confirmed by observing the contours of the von Mises stress within the cube.

### Preparation of the Brain Tissue Phantom

0.6% agarose gel was prepared to mimic the physical characteristics of the mouse brain. Agarose powder (A9539, Sigma‐Aldrich, Germany) and DI water were mixed and stirred for 1 h at 121 °C until the solution became transparent. The solution was then poured into a 3D printed mouse brain mold designed using fusion 360 and printed on Form 2 (Formlab, USA). It was then left to cool down at room temperature to allow for gel formation. The gel was freshly made and immediately used in each experiment.

### Rheological Analysis of the Brain Tissue Phantom

The storage modulus (*G'*) and loss modulus (*G''*) of the agarose gel brain tissue phantom were measured using a rheometer (MCR102; Anton Paar, Graz, Austria). After loading 200 µL of 0.4%, 0.5%, or 0.6% agarose gel, the sample was pressed using a flat plate with a diameter of 25 mm and a gap of 200 µm. Afterward, the sample was measured in a frequency sweep mode of 0.1–10.0 Hz at a constant shear strain of 1%.

### Insertion Experiment into the Brain Tissue Phantom

Each of the prepared brain phantoms was placed on the 10 mm diameter disk‐shaped load cell of a digital force gauge (DFG35‐0.12, Omega Engineering Inc., USA). The neural probes were fixed to the sample holder of the motorized Z‐stage and placed at a distance of 5 mm above the brain phantom surface. Next, the digital force gauge was zeroed to use as a baseline signal for the reference zero‐force level. After calibration, the probe was inserted into the prepared brain phantom at a speed of 0.1 mm s^−1^ to the target insertion depth of 1.85 mm. Force measurements began above the noise level (0.05 mN) and continued until the probe stopped moving. This entire experiment was conducted on an anti‐vibration table.

### Impedance Analysis for the Stability of the Lubricant Layer

The stability of the lubricant layer was monitored by measuring the impedance each time a LIPS neural probe was inserted into and removed from the brain phantom. The probe was repeatedly inserted into the prepared agarose gel brain phantom at a speed of 0.1 mm s^−1^ using a dip coater to a depth of 1.85 mm. Its removal was conducted at the same speed and EIS was performed to measure the impedance changes with a frequency sweep of 100 Hz to 100 kHz (SP‐200, Bio‐Logic, France).

### In Vitro Assay against Biological Substances

To make the aqueous protein solution, fluorescein isothiocyanate (FITC)‐conjugated albumin (A9771, Sigma‐Aldrich, Germany) was dissolved in phosphate‐buffered saline (PBS, 10 × 10^−3^ m, pH 7.4) to a final concentration of 1 mg mL^−1^. After rinsing the substrates with PBS to rehydrate the surfaces, they were immersed in six‐well plates that were then filled with 5 mL of aqueous protein solution, followed by incubation at 37 °C for 120 min. After incubation, the substrates were removed from the protein solution, gently washed three times with PBS, and fixed with 4% paraformaldehyde (*n* = 3 for each protein).

Mouse microglial cells SIM‐A9 (CRL‐3265, ATCC, USA) were cultured using Dulbecco's modified Eagle medium (DMEM) (30‐2006, ATCC, USA) supplemented with 5% heat‐inactivated horse serum (HS) (26050070, Gibco Molecular Probes, New Zealand), 10% heat‐inactivated fetal bovine serum (FBS) (S181H, Bio‐west, USA), and 1% penicillin‐streptomycin (PS) (LS202‐02, Welgene, Korea). The substrates were placed in a six‐well cell culture plate and the cells were seeded at 10^6^ cells cm^−2^. The cells were then incubated at 37 °C under 5% CO_2_. To prepare the cells for staining, the cells were fixed with 4% paraformaldehyde for 15 min and permeabilized with 0.5% Triton X‐100 for 5 min. After washing with PBS, Phalloidin conjugated to Alexa Fluor 488 (1:200, Abcam, USA) was used to stain actin filaments. The substrates were mounted in a Vectashield mounting medium containing 4′6‐diamidino‐2‐phenylindole (DAPI) (H‐1200, Vector Laboratories, UK) (*n* = 3).

### Animal Preparation and Surgery

All of the procedures involving the use of animals were approved by the Korea Institute of Science and Technology (KIST) in Seoul, Korea, and the procedures were conducted in accordance with the ethical standards stated in the Animal Care and Use Guidelines of KIST. The mice were provided by the animal facility in KIST. Adult male C57BL/6 mice (8–10 weeks old) were used in the study. The mice were housed in a cage under a 12:12 light‐dark cycle at the animal facility.

The mice were anesthetized with 4% isoflurane for induction and 2% isoflurane during surgery using an isoflurane vaporizer (SurgiVet Classic T3 vaporizer, Smiths Medical, Inc., Minneapolis, Minnesota, USA). The anesthetized mice were placed on a stereotaxic instrument (David Kopf Instruments, USA), and the hair and scalp of the mice were removed. After drilling five sites on the skull of each mouse, including the target site based on the atlas of Paxinos and Franklin,^[^ ^45^ ^]^ the four screws were tightly secured into the four holes on the skull except for the probe insertion region. After carefully removing the dure mater, the neural probe was inserted into the hippocampal CA3 region (−1.7; −2.0; −1.85, AP; ML; DV, in millimeters from the bregma). After placing the neural probe into the insertion site, it was fixed to the skull using screws on dental cement (Vertex Self Curing, Vertex Dental, Netherlands). Finally, to protect the neural probe from breaking by external impact, a custom crown was fixed that was manufactured using a 3D printer (Ultimaker 2+, Ultimaker, Netherlands) onto the skull using the dental cement.

### Electrophysiological Recording and Signal Analysis

To accurately compare the signal recording quality of coated and uncoated probes, neural activities from mice that had been anesthetized with 1% isoflurane using an isoflurane vaporizer were measured. Measurement of neural signals under anesthesia is free of electrical noise caused by mice's behavior, thus enabling an accurate comparison of recording quality between two probes. A commercialized signal recording system (RHD2000 Evaluation System with an RHD 16‐channel recording headstage, Intan Technologies, USA) was used to measure neural activities from the hippocampal neurons in anesthetized mice using two probes. The signals were filtered and recorded using Intan evaluation software for measuring action potentials from individual neurons (0.3–6.0 kHz of the bandpass filter). The signals from 16 electrodes at a sampling rate of 20 kS s^−1^ were collected. In the same way, the short‐term neural signal recording was performed around 12 h after surgery, while long‐term neural signal recording was performed once a week until no signal was detected for two consecutive weeks.

To classify recorded neural signals, a custom MATLAB spike‐sorting algorithm was used, which semi‐automatically sorted neural signals by the principal component analysis (PCA) and k‐means clustering. First, the threshold amplitude was set, and then spikes above threshold amplitude were automatically detected. Detected spikes were represented as PC1 and PC2 on the graph, and the optimal number of clusters for k‐means clustering was automatically determined by the elbow method. Finally, recorded neural signals were classified as individual neural signals by entering the optimal clustering number. The SNRs of the sorted signals were calculated by dividing the mean of the peak amplitude of the signals by three times the standard deviation of the background noise.^[^ ^42^ ^]^ Sorted signals were expressed on transient plots and heatmaps showing the firing rate over time, and bar plots were used for quantitative analysis of the signal recording quality comparison between two probes. The significance of the results was assessed with Student *t*‐tests using GraphPad Prism (Graphpad Software Inc., USA).

### Immunohistochemistry Analysis of the Foreign Body Response

The expression levels of the targeted proteins (GFAP and Iba1) were analyzed via immunohistochemistry (IHC) at time points of day 1, week 4, and week 12 (*n* = 3 per time point). A 4% paraformaldehyde (PFA) solution in PBS (pH 7.3) was pumped transcardially to anesthetize the mice for perfusion. Brains were extracted and fixed in 4% PFA solution overnight after removal of the probe. Paraffin‐embedded brains were sliced into 4 µm thick horizontal sections with an automated rotary microtome (RM2255, Leica, Germany) and washed using a solution of 0.3% Triton X‐100 and 3% bovine serum albumin (BSA) in PBS for 1 h. This was followed by overnight incubation in a solution of primary antibodies (goat anti‐GFAP 1:200 (ab53554, Abcam) and rabbit anti‐Iba1 1:500 (ab150136, Abcam) with 3% BSA in PBS at 4 °C. The sections were washed three times with PBS for 30 min and then stained with secondary antibodies (donkey anti‐goat antibodies labeled with Alexa Fluor 594 1:1000 (ab150136, Abcam) or goat anti‐rabbit antibodies labeled with Alex Fluor 488 1:1000 (ab150081, Abcam)) for 2 h at room temperature. After washing three times once more, the brain slices were mounted on glass microscope slides using Vectashield antifade mounting medium with DAPI (Vector Laboratories). A laser scanning confocal microscope (LSM 980, Carl Zeiss, Oberkochen, Germany) with a 20× objective lens was used for image acquisition of the brain slices under identical microscope settings, and the acquired images were analyzed with a customized MATLAB algorithm.

### Statistical Analysis

GraphPad Prism 8 software (Graphpad Software Inc., USA) was used to assess the statistical significance. The differences between groups were assessed via unpaired *t*‐tests and one‐way ANOVA tests.

## Conflict of Interest

The authors declare no conflict of interest.

## Supporting information

Supporting Information

## Data Availability

Research data are not shared.
