# Supplementary material for: A Lubricated Nonimmunogenic Neural Probe for Acute Insertion Trauma Minimization and Long‐Term Signal Recording
Source: Adv Sci (Weinh). 2021 Jun 3;8(15):2100231. doi: 10.1002/advs.202100231 (PMC8336494; doi:10.1002/advs.202100231)
Supplement: Supplementary file 1 — Supporting Information [file ADVS-8-2100231-s001.pdf]

© 2021 Wiley-VCH GmbH

ADVANCED SCIENCE
Open Access

Supporting Information

for *Adv. Sci.,* DOI: 10.1002/advs.202100231

A lubricated non-immunogenic neural probe for acute insertion trauma minimization and long-term signal recording

Yeontaek Lee †, Hyogeun Shin†, Dongwon Lee, Sungah Choi, Il-Joo Cho, Jungmok Seo**

Supporting Information

A lubricated non-immunogenic neural probe for acute insertion trauma minimization and long-term signal recording

Yeontaek Lee †, Hyogeun Shin†, Dongwon Lee, Sungah Choi, Il-Joo Cho, Jungmok Seo**

† These authors contributed equally to this work.
* Corresponding author.

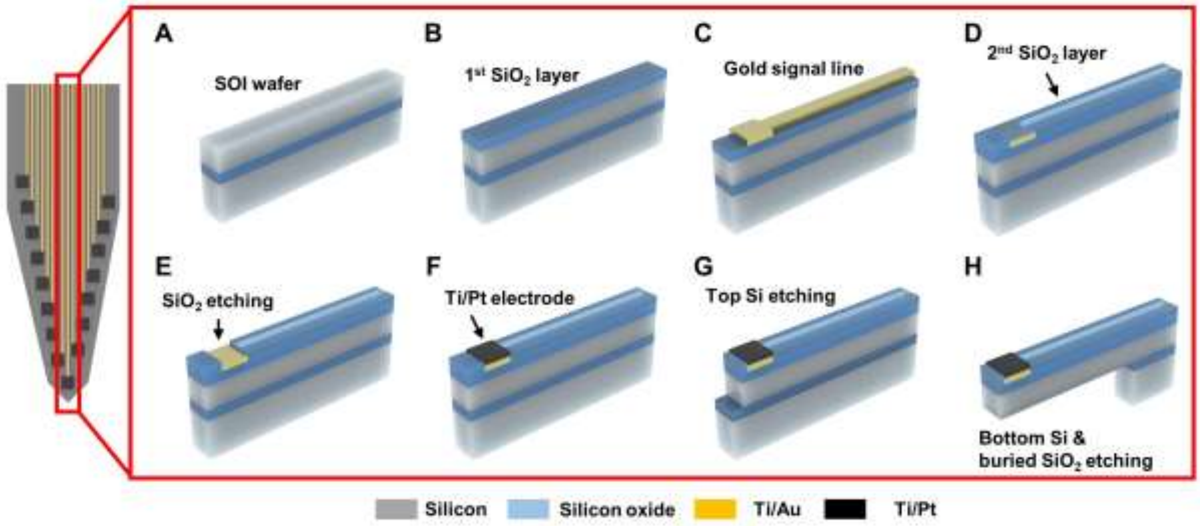

Figure S1. Schematic of the fabrication process of the neural probe and its black Pt electrodes. A, Preparation of a silicon-on-oxide wafer with a 40 μm-thick top silicon layer. **B**, 1st SiO_2 layer deposition (400 nm-thick). **C**, Gold signal line deposition and patterning. **D**, 2nd SiO_2 layer deposition (400 nm-thick). **E**, Reactive ion etching (RIE) of SiO_2 on the electrode sites. **F**, Ti/Pt (200 Å/1500 Å) deposition on the electrode sites. **G**, Deep RIE of the top silicon layer. **H**, Deep RIE of the bottom silicon layer and RIE of the buried SiO_2 layer.

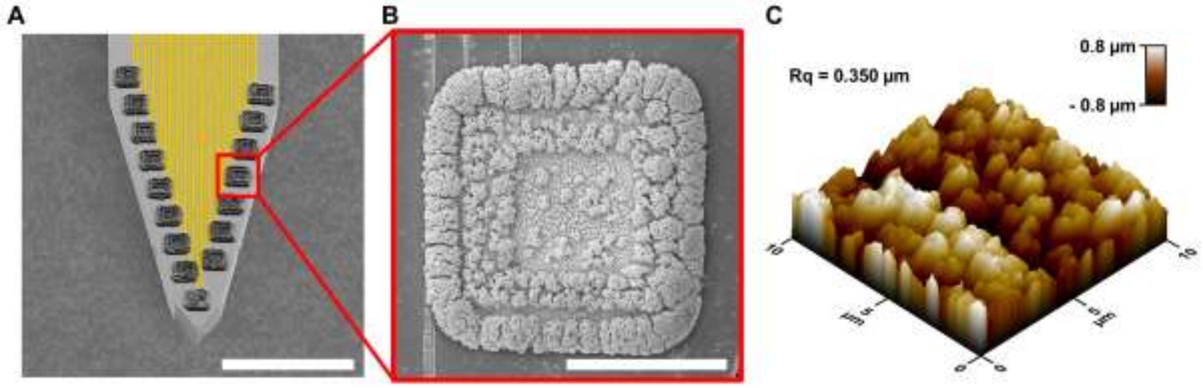

Figure S2. Visual confirmation of Pt black deposition on the electrodes on the neural probe shank. A, False-colored scanning electron microscopy (SEM) image of the surface-modified electrodes on the neural probe shank (scale bar, 100 µm). **B**, SEM image of Pt black deposited on the electrode surface for micro/nanostructure formation (scale bar, 10 µm). **C**, Atomic force microscopy image of the Pt black deposited electrode surface showing successful micro/nanostructure formation.

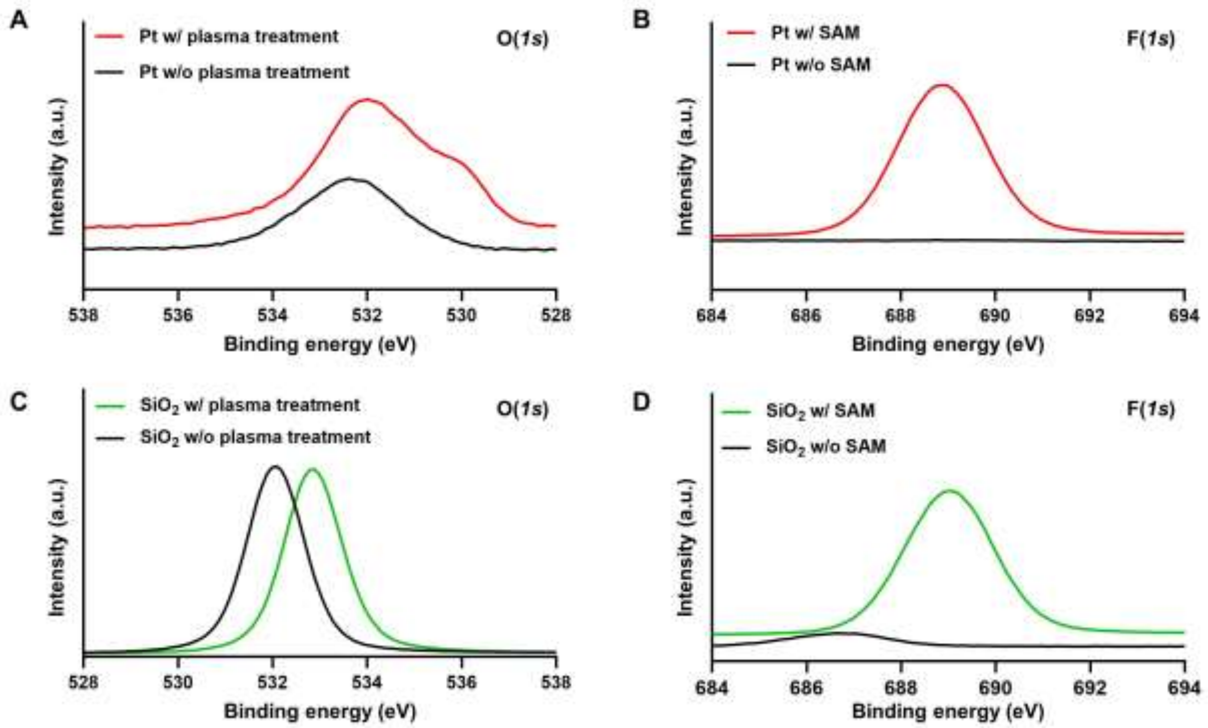

Figure S3. X-ray photoelectron spectroscopy (XPS) spectra of the substrate surface confirming changes in chemical modification. **A**, O 1s XPS spectra of the Pt black before and after O_2 plasma treatment. **B**, F 1s XPS spectra of the Pt black surface before and after self-assembled monolayer (SAM) formation. **C**, O 1s XPS spectra of the SiO_2 surface before and after O_2 plasma treatment. **D**, F 1s XPS spectra of the SiO_2 surface before and after SAM formation.

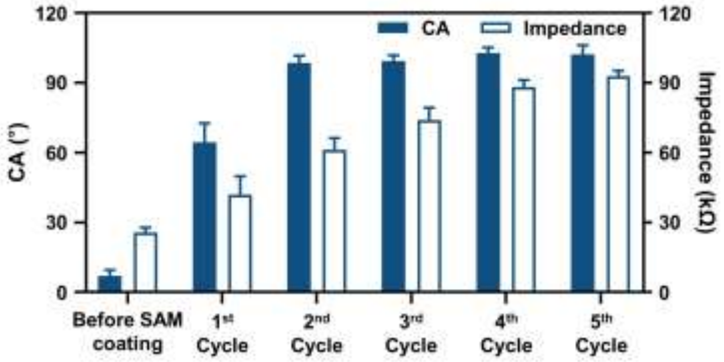

Figure S4. Contact angle measurements of and impedance at 1 kHz following each cycle of SAM coating formation. Data are presented as mean ± standard deviation. n = 5 for both CA and impedance measurement.

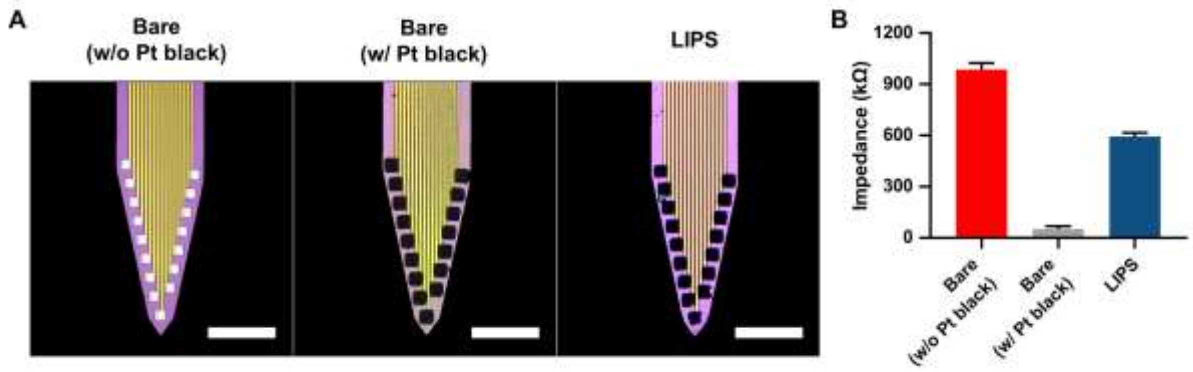

Figure S5. Impedance measurements following surface modification of neural probe electrodes. A, Optical microscopy images of bare uncoated neural probes (without and with Pt black) and a Lubricated Immune-stealthy Probe Surface (LIPS) neural probe (scale bars, 100 µm). **B**, Impedance measurements at 1 kHz from bare uncoated gold electrodes, bare uncoated gold electrodes onto which Pt black was deposited, and LIPS electrodes. Data are presented as mean ± standard deviation. n = 5.

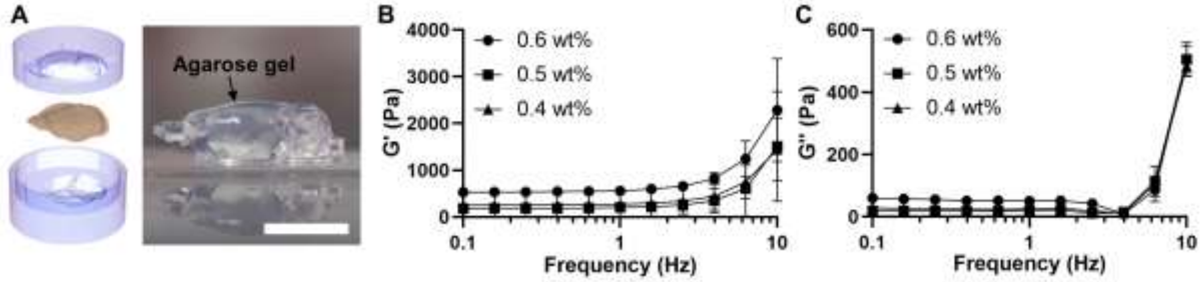

Figure S6. Brain phantom characteristics. A, Exploded top-to-bottom illustration of the brain phantom mold and optical image of the brain phantom product (scale bar, 5 mm). **B**,**C**, Storage modulus (G') **(B)** and loss modulus (G'') **(C)** measurements of 0.4, 0.5, and 0.6 wt% agarose gel brain phantoms for rheological analysis. Data are presented as mean ± standard deviation. (n = 5; n is the number of the sample)

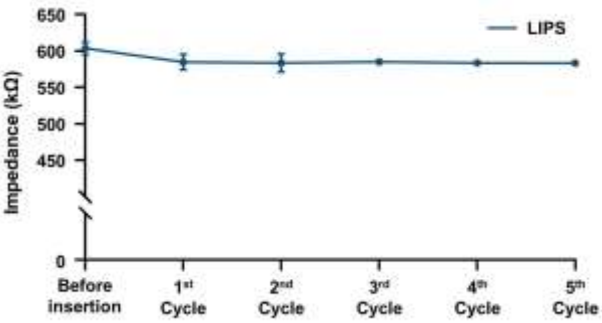

Figure S7. Impedance measurements at 1 kHz of the LIPS-coated probe electrodes following each cycle of probe insertion and extraction. Data are presented as mean ± standard deviation. (n = 3; n is the number of the repeated measurements)

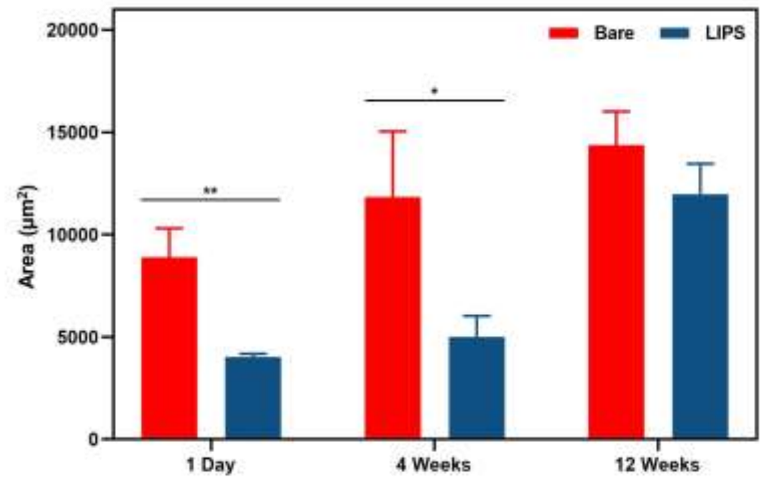

Figure S8. Tissue damage inflicted by probe insertion friction quantified as area. Data are presented as mean ± standard deviation. **P = 0.0040, n = 3 for bare and LIPS-coated probes on Day 1. *P = 0.0, n = 3 for bare and LIPS-coated probes at 4 weeks: n is the number of samples. *P < 0.05; **P < 0.01; ***P < 0.001; ****P < 0.0001; NS: no significant difference.
